# Aberrant Methylation Inactivates Somatostatin and Somatostatin Receptor Type 1 in Head and Neck Squamous Cell Carcinoma

**DOI:** 10.1371/journal.pone.0118588

**Published:** 2015-03-03

**Authors:** Kiyoshi Misawa, Yuki Misawa, Haruki Kondo, Daiki Mochizuki, Atsushi Imai, Hirofumi Fukushima, Takayuki Uehara, Takeharu Kanazawa, Hiroyuki Mineta

**Affiliations:** 1 Department of Otolaryngology/Head and Neck Surgery, Hamamatsu University School of Medicine, Shizuoka, Japan; 2 Department of Head and Neck, Cancer Institute Hospital of Japanese Foundation for Cancer Research, Tokyo, Japan; 3 Department of Otorhinolaryngology, Head and Neck Surgery, Graduate school of Medicine, University of the Ryukyus, Okinawa, Japan; 4 Department of Otolaryngology/Head and Neck Surgery, Jichi Medical University, Shimotsuke, Japan; Queen Mary University of London, UNITED KINGDOM

## Abstract

**Purpose:**

The aim of this study was to define somatostatin (*SST*) and somatostatin receptor type 1 (*SSTR1*) methylation profiles for head and neck squamous cell carcinoma (HNSCC) tumors at diagnosis and follow up and to evaluate their prognostic significance and value as a biomarker.

**Methods:**

Gene expression was measured by quantitative RT-PCR. Promoter methylation status was determined by quantitative methylation-specific PCR (Q-MSP) in HNSCC.

**Results:**

Methylation was associated with transcription inhibition. *SST* methylation in 81% of HNSCC tumor specimens significantly correlated with tumor size (*P* = 0.043), stage (*P* = 0.008), galanin receptor type 2 (*GALR2*) methylation (*P* = 0.041), and tachykinin-1 (*TAC1*) (*P* = 0.040). *SSTR1* hypermethylation in 64% of cases was correlated with tumor size (*P* = 0.037), stage (*P* = 0.037), *SST* methylation (*P* < 0.001), and expression of *galanin* (*P* = 0.03), *GALR2* (*P* = 0.014), *TAC1* (*P* = 0.023), and tachykinin receptor type 1 (*TACR1*) (*P* = 0.003). *SST* and *SSTR1* promoter hypermethylation showed highly discriminating receiver operator characteristic curve profiles, which clearly distinguished HNSCC from adjacent normal mucosal tissues. Concurrent hypermethylation of *galanin* and *SSTR1* promoters correlated with reduced disease-free survival (log-rank test, *P* = 0.0001). Among patients with oral cavity and oropharynx cancer, methylation of both *SST* and *SSTR1* promoters correlated with reduced disease-free survival (log-rank test, P = 0.028). In multivariate logistic-regression analysis, concomitant methylation of *galanin* and *SSTR1* was associated with an odds ratio for recurrence of 12.53 (95% CI, 2.62 to 59.8; *P* = 0.002).

**Conclusions:**

CpG hypermethylation is a likely mechanism of *SST* and *SSTR1* gene inactivation, supporting the hypothesis that *SST* and *SSTR1* play a role in the tumorigenesis of HNSCC and that this hypermethylation may serve as an important biomarker.

## Introduction

Squamous cell carcinoma of the head and neck (HNSCC) is the sixth most frequent type of cancer. [1] The use of targeted drugs is an increasingly adopted anticancer strategy; the application of epidermal growth factor receptor (EGFR)-specific antibodies combined with radiotherapy is a prominent example. However, despite high expression of EGFR in HNSCC, EGFR inhibitor monotherapy has only a modest impact on survival. [2] Recently, a tumor suppressor role for neuropeptides that is mediated via the autocrine and/or paracrine systems has been proposed. [3] Our findings suggest that simultaneous methylation of *galanin*, galanin receptor type 1 (*GALR1*), and *GALR2* genes occurs in a subset of HNSCC and may be used as a prognostic marker. [4,5] Somatostatin (SST) was first identified as a growth hormone release-inhibitory factor in ovine hypothalamus in 1973. [6] Its main functions involve regulating endocrine and exocrine secretion, modulating motor activity, and inhibiting gastrin-stimulated gastric acid secretion in the gastrointestinal tract. [7] In recent years, several studies have suggested that *SST* functions as a tumor suppressor gene and possesses potent antitumor and antisecretory activities in several human cancers in vitro and in vivo. [7] *SST* suppresses tumor growth through distinct mechanisms that involve inhibition of growth factors and hormones, reduction in vascularization, and regulation of the immune system. [8] Hypermethylation of *SST* has been described in esophageal cancer, [7] gastric cancer, [9] colon cancer, [10] and renal cancer. [11] Promoter hypermethylation concomitant with transcriptional silencing of *SSTR1* expression has been detected in EBV-positive gastric cancer cells.[12] Despite our understanding of gastrointestinal tract cancer, hypermethylation in head and neck cancer remains to be explored. The purpose of this study was to first define a *SST* and *SSTR1* methylation profile in HNSCC tumors analyzed at the time of diagnosis and then to evaluate its value as a prognostic and recurrence biomarker.

Neuroendocrine peptides play essential roles in the regulation of gastrointestinal endocrine and exocrine secretion, motility, and mucosal immunity. Moreover, some neuroendocrine peptides, including *SST*, have been implicated in the modulation of human tumorigenesis by both direct and indirect means. The current findings provide novel direct epigenetic evidence in human patients for the involvement of *SST* in the process of human tumor suppression. [10] Kharmate et al. reported that SSTR1 controls EGF-mediated cell survival via dissociation of an ErbB heteromeric complex. [13] Others recently reported that both SSTRs and ErbBs activate the MAPK pathway, as SST-induced MAPK activation results in delayed cell cycle progression, whereas EGF activation promotes proliferation. [14] Therefore, detection of aberrant expression of SST/SSTR1 may be of potential use as a marker for selecting HNSCC patients who could benefit from additional targeted therapies.

To test this hypothesis, we studied methylation of the *SST* and *SSTR1* promoters by Q-MSP in 100 head and neck tumors of differing primary sites. More recently, data from our laboratory have shown that the *galanin*, *GALR1*, *GALR2*, *TAC1*, and *TACR1* promoters are methylated in HNSCC. [15,16] Therefore, we hypothesized that neuropeptide genes and receptor genes might be inactivated via promoter hypermethylation in human head and neck cancers, and that hypermethylation of these genes is an important event in the genesis of HNSCC. Moreover, we discovered a unique inverse relationship between *SST* and *SSTR1*-promoter hypermethylation and other neuropeptide genes.

## Materials and Methods

### Tumor samples and cell lines

Tumor specimens in an original cohort were obtained at surgery from 100 primary HNSCC samples. All patients were treated at the Department of Otolaryngology, Hamamatsu University School of Medicine, between 1977 and 1995. Clinical information including age, sex, tumor site, smoking status, alcohol exposure, tumor size, lymph node status, and stage grouping were obtained from the clinical records. The mean age was 63.6 years (range 39–93 years), and the male:female ratio was 78:22. The primary tumor was located in the oral cavity (n = 34), the hypopharynx (n = 24), the larynx (n = 20), the oropharynx (n = 11), and the paranasal cavity (n = 11). Matched pairs of head and neck tumor tissues and adjacent normal mucosal tissues were obtained from the surgical specimens of 36 patients for initial methylation screening between 2008 and 2011. The normal oropharynx samples were obtained from chronic tonsillitis patients after tonsillectomy. All patients provided written informed consent under a protocol approved by the Institutional Review Boards at the Hamamatsu University School of Medicine. DNA and cDNA were derived from12 UM-SCC cell lines established from patients at the University of Michigan. Fibroblasts from the original tumor specimen were used as the source of normal somatic DNA. [17] Nonmalignant cells from the donors of UM-SCC cell lines have the same number [e.g., UM-SCC-6 and UM-6F (fibroblasts)]. Other control cells included normal human keratinocytes (NHK).[18] UM-SCC cell lines were kindly provided by Dr. Thomas E. Carey of the University of Michigan and were validated by genotyping in his laboratory. [4,5]

### Bisulfite modification and quantitative methylation-specific PCR (Q-MSP)

Genomic DNA was extracted with the Wizard Genomic DNA Purification Kit (Promega, Madison, WI). Bisulfite modification of genomic DNA was performed as reported in a previous study. [4] Promoter methylation of *SST* was measured by quantitative methylation-specific PCR (Q-MSP) with the TaKaRa Thermal Cycler Dice TM Real-Time System TP800 (TaKaRa, Tokyo, Japan). Q-MSP primers for methylated DNA were Q-MSP-*SST*-F (5ʹ- GGG GCG TTT TTT AGT TTG ACG T-3ʹ) and Q-MSP-*SST*-R (5ʹ-AAC AAC GAT AAC TCC GAA CCT CG-3ʹ), Q-MSP-*SSTR1*-F (5ʹ- CGG GTG CGC GAG GAG AAA GTT-3ʹ) and Q-MSP-*SSTR1*-R (5ʹ- TAG TTC GGG TAG TTG CGG CGA A-3ʹ), and Q-MSP-*ACTB*-F (5ʹ-TGG TGA TGG AGG AGG TTT AGT AAG T-3ʹ) and Q-MSP-*ACTB*-R (5ʹ-AAC CAA TAA AAC CTA CTC CTC CCT TAA-3ʹ). Q-MSP was carried out and the normalized methylation value (NMV) was defined as described previously. [4] To analyze the methylation status of *TAC1* [16], *TACR1* [16], *Galanin* [15], *GALR1* [4] and *GALR2* [5] primers, conditions, as described previously, were used.

### Quantitative RT-PCR of *SST* and *SSTR1*

Total RNA was isolated with the RNeasy Mini Kit (QIAGEN, Hilden, Germany) and treated with RNase-Free DNase (QIAGEN). cDNA was generated from DNase-treated total RNA by using random primers (Invitrogen, Carlsbad, CA) with Superscript II reverse transcriptase (Invitrogen). The primer sequences were as follows: *SST* forward, 5ʹ-CCA GAC TCC GTC AGT TTC TGC A-3ʹ; *SST* reverse, 5ʹ-CAT CAT TCT CCG TCT GTT TGG GTT-3ʹ [10]; *SSTR1* forward, 5ʹ- TCT GCG CGA AGA TCG TCA AC-3ʹ; *SSTR1* reverse, 5ʹ- GCG GCT CTG GAC TGG TAA ATG-3ʹ (TaKaRa, Tokyo, Japan); *GAPDH* forward, 5ʹ-GCA CCG TCA AGG CTG AGA AC-3ʹ; and *GAPDH* reverse, 5ʹ-TGG TGA AGA CGCCAG TGG A-3ʹ. To analyze the expression of *SSTR2*, *SSTR3*, *SSTR4*, and *SSTR5*, we used previously described primers and conditions. [19] Quantitative-RT-PCR was performed on the TaKaRa TP800 system. Quantitative RT-PCR was carried out as described previously. [4]

### Statistical analysis

Receiver-operator characteristic (ROC) curves were generated by using the NMVs for the 36 HNSCC and 36 adjacent normal mucosal tissues. The area under the ROC curve identified optimal sensitivity and specificity levels at which normal tissues could be distinguished from HNSCC tissues, and corresponding NMV thresholds were calculated for *SST*. The cutoff value determined from this ROC curve was applied to determine the frequency of *SST* methylation.

To determine the overall rate of methylation in individual samples, we used the Methylation Index (MI). [20,21] The MI for each sample was defined as the ratio of the number of methylated genes to the number of genes tested (seven in this study; *SST*, *SSTR1*, *TAC1*, *TACR1*, *Galanin*, *GALR1* and *GALR2*). Global Methylation Index (GMI) was calculated by taking the sum of gene promoter hypermethylation events in each tumor divided by the number of genes examined (eight in this study; *p16*, *RASSF1A*, *E-cadherin*, *H-cadherin*, *MGMT*, *DAPK*, *DCC*, and *COL1A2*). Mean differences in MI and GMI by histological types and cancer risk factors were examined by employing stratified analysis of variance (Student’s *t*-test).

For frequency analysis in contingency tables, associations between variables were analyzed by Fisher’s exact test. Comparisons and tests for statistical significance in the colony formation assay were made with the Student’s *t*-test. The disease-free interval was measured from the date of treatment to the date when locoregional recurrence or distant metastasis was diagnosed. Disease-free survival (DFS) probabilities were estimated by the Kaplan-Meier method, and the log-rank test was applied to assess the significance of differences between actuarial survival curves. Multivariate logistic-regression analysis, which involved age, sex, smoking status, alcohol intake, stage grouping, and methylated genes, was used to identify the predictive value of the prognostic factors. [1,22] P values are two-tailed and significance was determined as *P* < 0.05. All statistical analyses were performed with StatMate IV (ATMS Co. Ltd., Tokyo, Japan).

## Results

### UM-SCC cell lines

**Fig 1:**
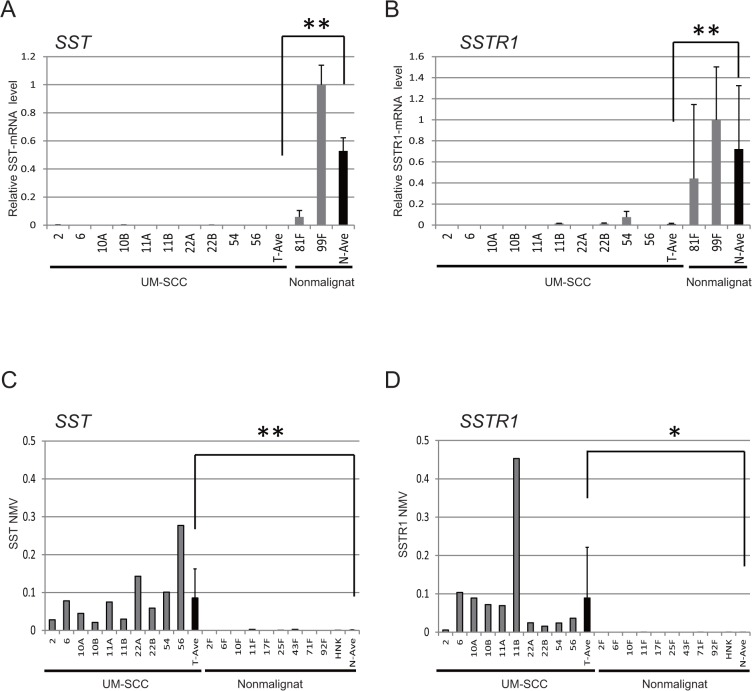
Diagrammatic representation of *SST* methylation analysis by quantitative-MSP, expression analysis by quantitative-RT-PCR, and bisulfite sequencing analysis in UM-SCC cell lines. **(A)** Relative mRNA expression of *SST* revealed lower expression in cancer cell lines than in normal fibroblasts (*P* < 0.01). The housekeeping gene *GAPDH* was run as a control for RNA integrity. **(B)** Relative mRNA expression of *TACR1* revealed lower expression in cancer cell lines than in normal fibroblasts (*P* < 0.01). **(C)** Mean *SST* NMV was significantly higher in cancer cell lines (*P* < 0.001). **(D)** Representative examples of quantitative-MSP of *TACR1* in cancer and normal fibroblasts and keratinocytes; there was a significant difference in NMV (*P* < 0.05).

Quantitative RT-PCR of *SST1* and *SSTR1* transcripts from 10 UM-SCC cell lines revealed lower expression in cancer cell lines than in normal fibroblasts (*P* < 0.01, **Fig. 1A, 1B**). Q-MSP technology indicated a significantly increased NMV of *SST1* promoter methylation in cancer cell lines versus normal fibroblasts and keratinocytes (*P* < 0.01, **Fig. 1C**). The NMV of *SSTR1* was significantly higher in UMSCC than in normal fibroblasts and keratinocytes (*P* < 0.05, Student’s *t*-test) (**Fig. 1D**).

### Matched pairs of head and neck tumors and adjacent normal mucosal tissues

**Fig 2:**
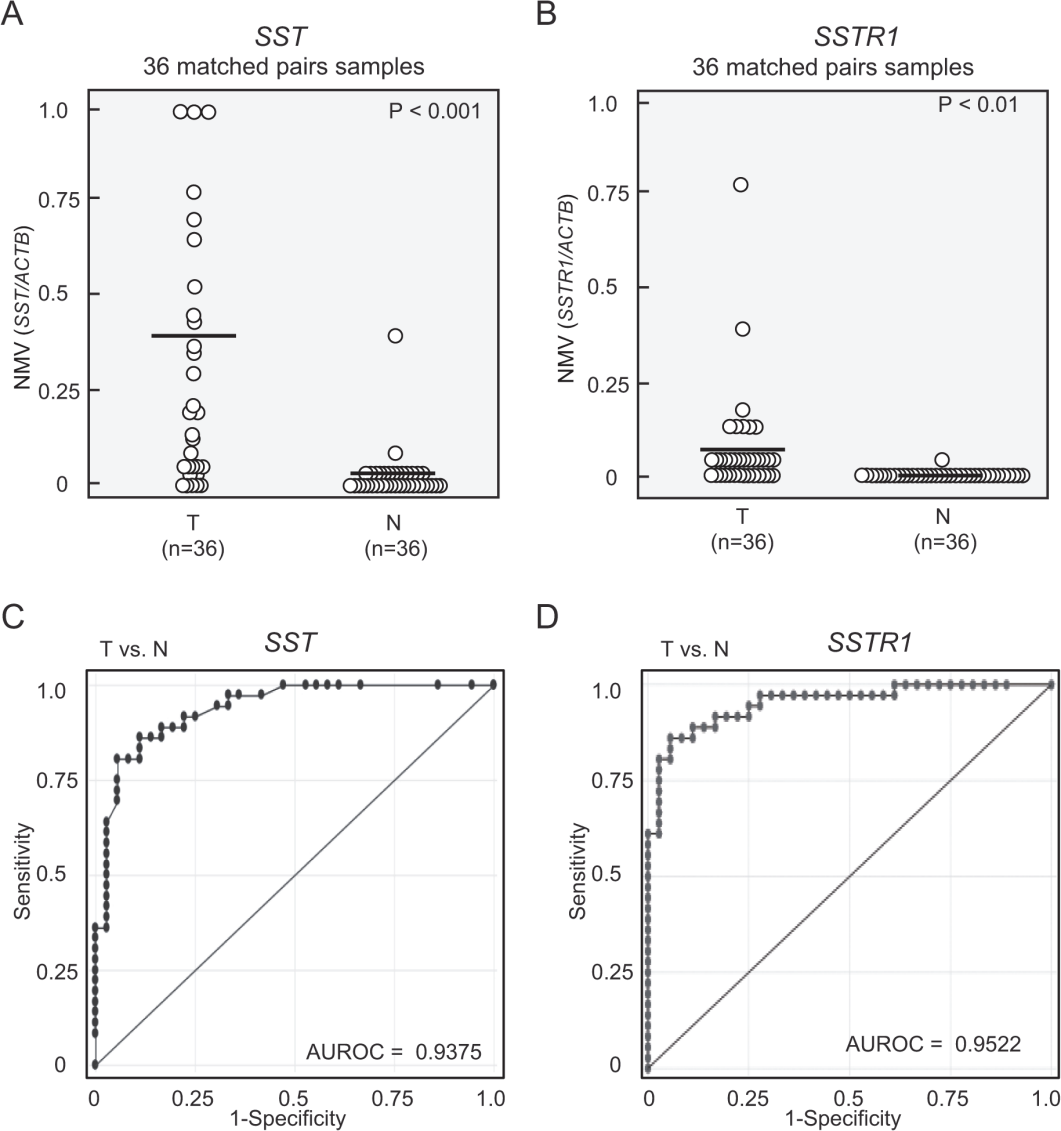
Hypermethylation patterns in matched pairs of head and neck tumors and adjacent normal mucosal tissues. **(A)** *SST* NMVs of head and neck tumors were significantly higher than those of paired adjacent normal mucosal tissues (*P* < 0.001). **(B)** A higher frequency and quantity of *SSTR1* methylation was noted in head and neck tumors than in matched normal mucosal tissues (*P* < 0.01). **(C)** The area under the ROC curve (AUROC) value for the *SST* gene was 0.9375. At the cutoff value of 0.046, sensitivity was 80.6% and specificity was 94.4%. **(D)** The AUROC value for the *SSTR1* gene was 0.9522. At the cutoff value of 0.012, sensitivity was 61.1% and specificity was 100%.

*SST* and *SSTR1* promoter methylation status were analyzed by Q-MSP in 36 cancerous and paired noncancerous mucosa. *SST* and *SSTR1* methylation levels were significantly higher in primary HNSCCs than in noncancerous mucosal tissues (median NMV = 0.331 versus 0.021, *P* < 0.001, and median NMV = 0.067 versus 0.001, *P* < 0.01, Wilcoxon matched-pairs test and paired Student’s *t*-test; **Fig. 2A, 2B**). *SST* and *SSTR1* promoter hypermethylation showed highly discriminative ROC curve profiles, which clearly distinguished HNSCC from normal mucosal tissues (AUROC = 0.9375, AUROC = 0.9522, respectively). ROC curves with corresponding areas under the ROC curve for target genes in HNSCC versus normal mucosal tissues is shown in Fig. 2C and 2D. The cutoff NMVs for *SST* (0.041) and *SSTR1* (0.012) were chosen from the ROC curves for high sensitivity and > 95% specificity (**Fig. 2C, 2D**).

### Clinicopathologic characteristics of 100 primary HNSCC samples

We classified a specimen as methylated when the NMV exceeded the cutoff value. The *SST* promoter was methylated in 81 of 100 (81%) cases (**Table 1**). The *SSTR1* promoter was methylated in 64 of 100 (64%) cases (**Table 1**). There was a significant correlation between the methylation status of the *SST* and *SSTR1* promoters (*P* < 0.001). Methylation of *SST* was associated with several clinicopathologic factors, including tumor size (*P* = 0.043), stage (*P* = 0.008), *GALR2* methylation (*P* = 0.041), *TAC1* methylation (*P* = 0.040), and *DAPK* methylation (*P* = 0.012) (**Table 1**and **S1 Table**). *SSTR1* methylation was significantly correlated with tumor size (*P* = 0.037), stage (*P* = 0.037), *galanin* (*P* = 0.030), *GALR2* methylation (*P* = 0.014), *TAC1* methylation (*P* = 0.023), *TAC1R* methylation (*P* = 0.003), *H-cadherin* methylation (*P* = 0.007), *MGMT* methylation (*P* = 0.001), *DAPK* methylation (*P* = 0.001) and *DCC* methylation (*P* = 0.045) (**Table 1**and **S1 Table**).

**Table 1:** SST and SSTR1 Genes Methylation Status in HNSCC Primary Samples.

Characteristics (n = 100)		*SST*			*SSTR1*		
		Present (81)	Absent (19)	P-value†	Present (64)	Absent (36)	P-value†
Age	70 and older (29)	24	5	1	18	11	1
	Under 70 (71)	57	14		46	25	
Gender	Male (78)	65	13	1	51	27	1
	Female (22)	16	6		13	9	
Smoking status	Smoker (66)	53	13	1	45	21	1
	Nonsmoker (34)	28	6		19	15	
Alcohol exposure	Ever (60)	49	11	1	39	21	1
	Never (40)	32	8		25	15	
Tumor size	T1–2 (52)	38	14	0.043	28	24	0.037
	T3–4 (48)	43	5		36	12	
Lympho-node status	N0 (46)	34	12	0.126	26	20	0.21
	N+ (54)	47	7		38	16	
Stage	I-III (49)	34	15	0.008	26	23	0.037
	IV (51)	47	4		38	13	
Recurrence events	Positive (47)	37	10	0.618	32	15	0.532
	Negative (53)	44	9		32	21	
*SST* methylation	Yes (81)	-	-	-	60	21	<0.001
	No (19)	-	-	-	4	15	
*Galanin* methylation	Yes (25)	23	2	0.185	21	4	0.030
	No (75)	58	17		43	32	
*GALR1* methylation	Yes (38)	33	5	0.3	29	9	0.055
	No (62)	48	14		35	27	
*GALR2* methylation	Yes (33)	31	2	0.041	27	6	0.014
	No (67)	50	17		37	30	
*TAC1* methylation	Yes (49)	44	5	0.040	37	12	0.023
	No (51)	37	14		27	24	
*TACR1* methylation	Yes (34)	30	4	0.186	29	5	0.003
	No (66)	51	15		35	31	

†Fisher’s exact probability test

### Comparison of MI among selected epidemiologic and clinical characteristics

**Fig 3:**
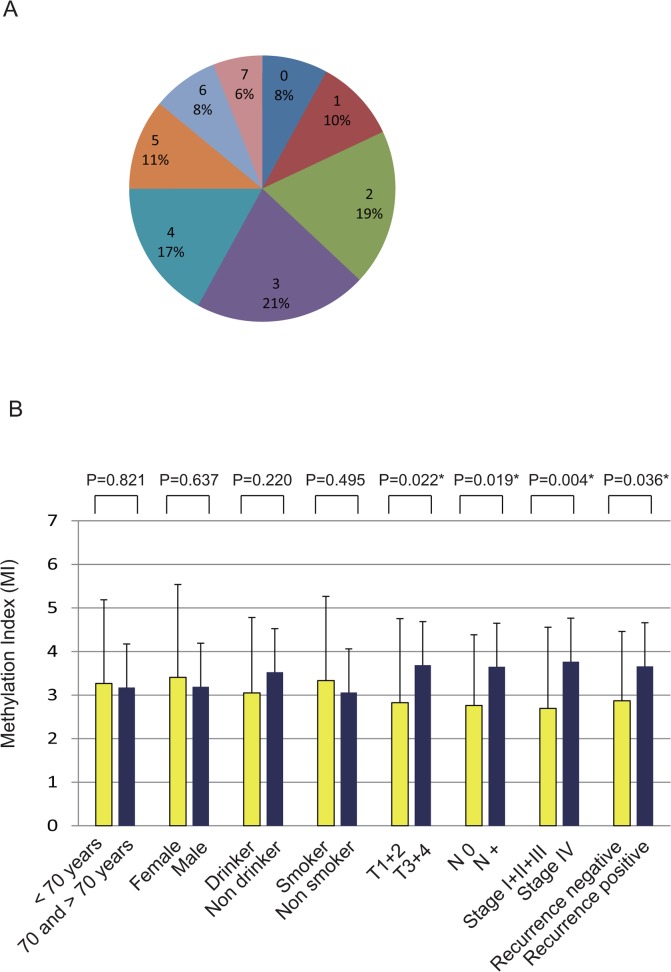
Gene promoter methylation analysis by quantitative-MSP in 100 primary HNSCC samples. **(A)** Percentage of patients with epigenetic alternation in *SST*, *SSTR1*, *TAC1*, *TACR1*, *galanin*, *GALR1*, and *GALR2* genes. **(B)** Comparison of MI among selected epidemiologic and clinical characteristics.

A representative methylation analysis for *SST*, *SSTR1*, *TAC1*, *TACR1*, *Galanin*, *GALR1*, and *GALR2* in tumors is shown in Fig. 3A. Fifty-eight percent (58 of 100) of the tumors included 0 to 3 hypermethylated genes: 17% had 4 hypermethylated genes, 11% had 5 hypermethylated genes, and 14% had 6 or 7 hypermethylated genes (**Fig. 3A**). We evaluated MI using promoter methylation of *SST*, *SSTR1*, *TAC1*, *TACR1*, *Galanin*, *GALR1*, and *GALR2*. Hypermethylation of MI was significantly associated with tumor size (*P* = 0.022), lymph-node status (*P* = 0.019), stage (*P* = 0.004), and recurrence events (*P* = 0.036). No differences were noted with regard to age at onset, gender, alcohol exposure, or smoking status (**Fig. 3B**). Based on data from continuous marker methylation analyses, GMI of *p16*, *RASSF1A*, *E-cadherin*, *H-cadherin*, *MGMT*, *DAPK*, *DCC*, and *COL1A2* were not correlated with any of the characteristics (**S1 Fig.**).

### Prognostic Value of the *SST*, *SSTR1*, and Other Genes

**Fig 4:**
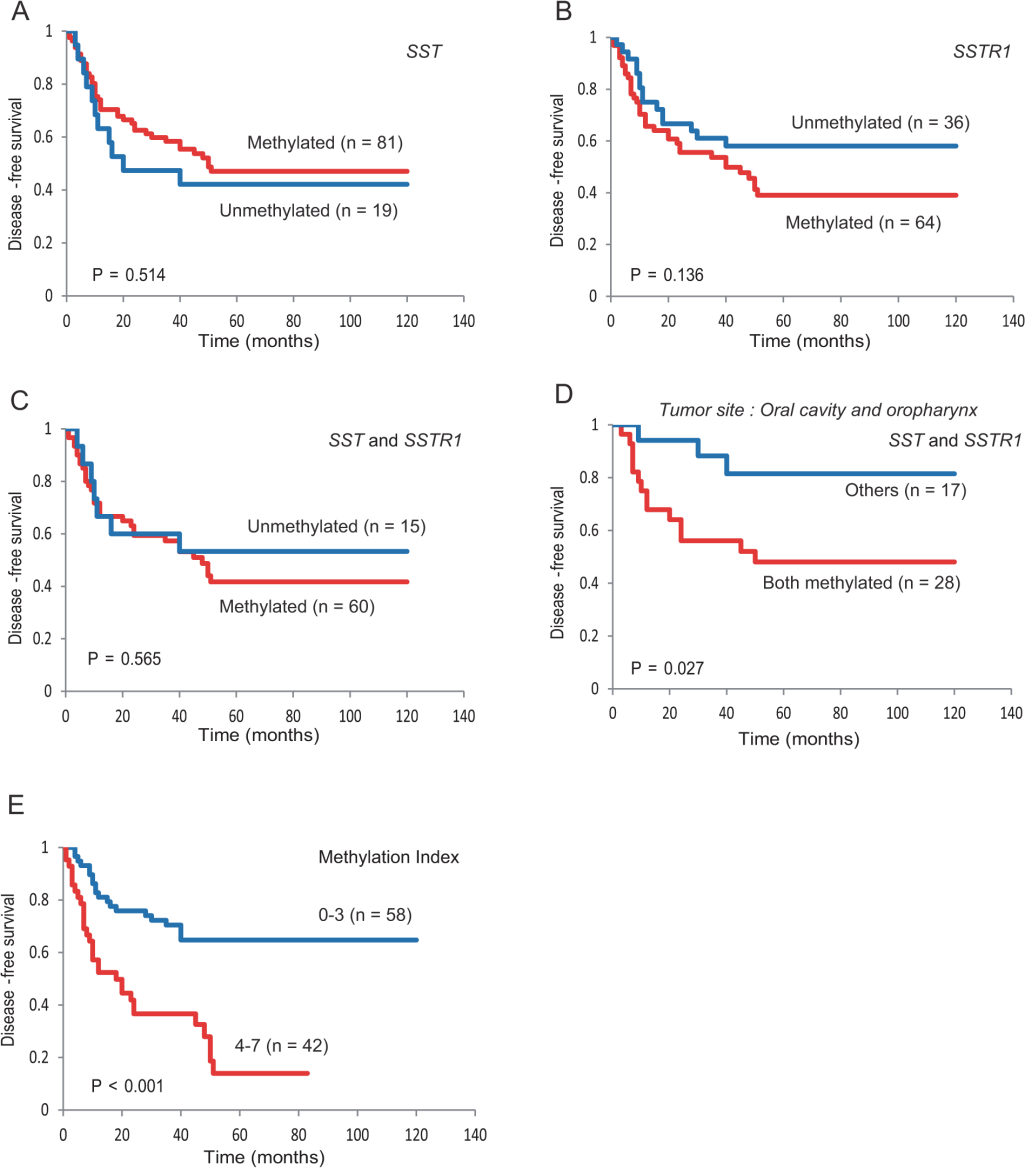
Kaplan-Meier survival curves for patients with HNSCC. Disease-free survival by **(A)** *SST* methylation status, **(B)** *SSTR1* methylation status, **(C)** *SST* and *SSTR1* methylation status, **(D)** *SST* and *SSTR1* methylation status in oral cavity and oropharynx patients, and **(E)** number of MI. Disease-free survival was briefer in patients with MI (4–7) than in those with MI (0–3) methylation (*P* < 0.001, Log-rank test). Blue line, patients without methylation; red line, patients with methylation.

Kaplan-Meier plots indicated that methylation of *SST*, *SSTR1*, and other genes in the patients’ tumors were related to the duration of DFS. *SST* methylation (log-rank test, *P* = 0.514) and *SSTR1* methylation (log-rank test, *P* = 0.136) were not associated with any difference in DFS (**Fig. 4A, 4B**). Methylation of both *SST* and *SSTR1* was not associated with an altered DFS rate when compared with samples harboring low levels of methylation (41.7% versus 53.3%, log-rank test, *P* = 0.565) (**Fig. 4C**). Among patients with oral cavity and oropharynx cancer, the disease-free survival rate in the group of patients with both *SST* and *SSTR1* methylation was 48.1% as compared with 81.4% in the other groups (log-rank test, *P* = 0.028) (**Fig. 4D**). The DFS was lower in the MI (4–7) methylated genes group than in the MI (0–3) methylated genes group (14.0% versus 64.7%, respectively; log-rank test, *P* < 0.001) (**Fig. 4E**). The DFS of the both *TAC1* and *SSTR1* methylation group was significantly higher than that of the no methylation group (log-rank test, *P* = 0.011) (**S2A Fig.**). Methylation of both *galanin* and *SSTR1* was associated with a DFS rate of 0% versus 59.0% in the absence of methylation (log-rank test, *P* = 0.0001) (**S2B Fig.**). Patients in which *GALR2* and *SSTR1* were not methylated survived significantly longer than those in which both genes were methylated (log-rank test, *P* = 0.005) (**S2C Fig.**). The DFS of the both *SSTR1* and *GALR1* methylation group was significantly higher than that of the no methylation group (log-rank test, *P* = 0.022) (**S2D Fig.**).

**Fig 5:**
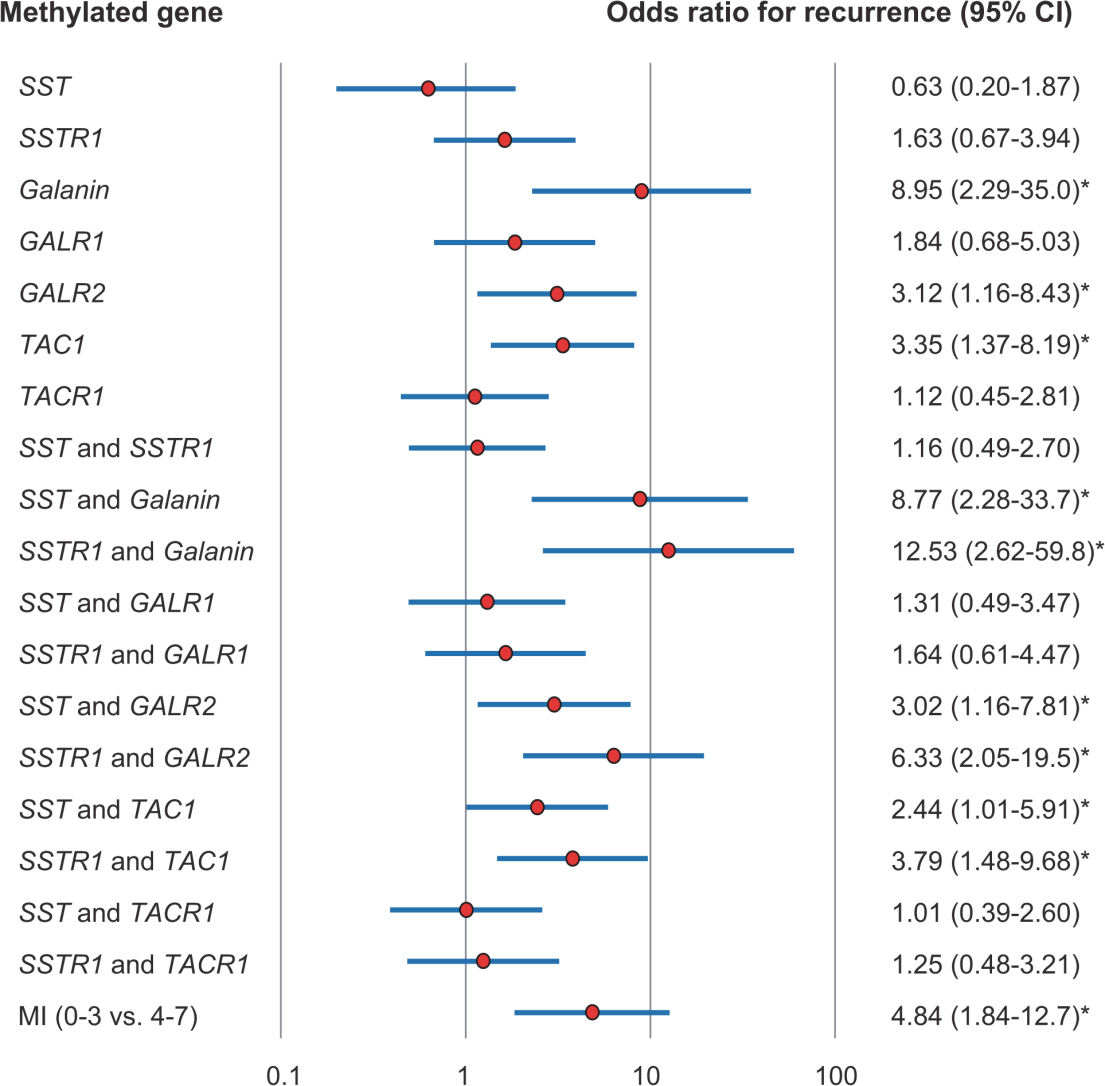
Odds ratios for recurrence based on multivariate logistic-regression adjusted for stage (I, II, III vs. IV), age (65 and older vs. <65), sex, alcohol exposure, and smoking status. Methylation of *SSTR1* and *galanin* in the primary tumor was associated with the most significant odds ratios of recurrence.

Multivariate logistic-regression analysis showed the estimated odds of recurrence associated with methylation of *SST*, *SSTR1*, and other genes. Methylation of either *SST* or *SSTR1* was associated with an elevation in the odds of recurrence that was not significant. Patients with *SSTR1* and *galanin* methylation had a highest odds ratio for recurrence (OR = 12.53, 95% CI, 2.62 to 59.81; *P* = 0.002) (**Fig. 5**). When MI was tested in the multivariate logistic-regression analysis, MI (0–3 vs. 4–7) was independently predictive of DFS after adjustment for stage (**Fig. 5)** and GMI **(S3 Fig.**).

## Discussion

Recent advances in molecular biology have made it possible to apply new strategies, such as gene therapy and molecular targeted therapy for cancer treatment. [23] However, in comparison to lesions such as breast, renal and colorectal carcinoma, HNSCC treatments are less advanced. [24] GPCRs belong to a superfamily of cell surface signaling proteins with a pivotal role in many physiological functions and multiple diseases, including cancer development and metastasis. [25] In recent years, *SST* and the somatostatin receptor (which belongs to the GPCR family) have been identified as tumor suppressor genes that possess potent antitumor and antisecretory activities in several human cancers in vitro and in vivo. [8] Head and neck tumor specimens expressed *SSTR1*, *SSTR2*, *SSTR4*, and *SSTR5*, whereas *SSTR3* mRNA expression was low. [26] A similar loss of *SSTR1* and *SSTR2* protein in normal versus malignant tissue has been observed in laryngeal lesions. [27] In our tumor cell series and normal oropharynx samples (NOS), the profiles of somatostatin and somatostatin receptor mRNAs were consistent with those reported in previous studies **(S4 Fig.**).

Consistent with this, a recent study showed that *SST* promoter hypermethylation is common in human esophageal adenocarcinoma, gastric cancer, and colon cancer. [7,9,10] Zhao et al. reported that ectopic expression of *SSTR1* in gastric cancer cell lines, which exhibit hypermethylation and express no *SSTR1* mRNA, significantly suppressed cell growth in culture conditions and reduced tumor size in nude mice. [12] Furthermore, methylation-dependent regulation of *SST* and *SSTR1* expression is observed in the chick embryonic liver during the developmental stages. [28] These findings provide a foundation for further studies on the role of neuropeptide genes and their receptors in carcinogenesis, and their potential utility as biomarkers for many types of tumors. Hypermethylation of *TAC1* has been described in esophageal cancer, gastric cancer, [29] colon cancer, [10] and breast cancer. [30] Overall patient survival is correlated with *TAC1* methylation status in esophageal squamous cell carcinoma, but not in esophageal adenocarcinoma. [31] Our preliminary analysis showed that silencing of the *TAC1* gene by methylation may be a critical event in tumor progression of HNSCC and that *TAC1* promoter methylation was associated with reduced overall survival rates.[16] Furthermore, the methylation of galanin significantly correlated with *GALR1* and *GALR2* methylation and reduced DFS. [4,5,15] The methylation of the gene pair of galanin and *GALR1* in the primary tumor was associated with the most significant odds ratio of recurrence, [15] while another study concluded that *GALR1* induces cell cycle arrest, and *GALR2* induces both cell cycle arrest and apoptosis in HNSCC following galanin treatment. [24] this study shows that aberrant DNA methylation of *SST* and *SSTR1* is not associated with prognosis.

Certain combinations of DNA hypermethylation patterns can be used as highly sensitive biomarkers, and can be used to identify cancer cells or to predict tumor progression in prostate and lung cancers, respectively. [32] [33] The methylation status of glutathione S-transferase pi 1 (*GSTP1*) was strongly associated with disease outcome in men with suspected prostate cancer. [34] Broch et al. reported that methylation of *p16* and *H-cadherin* was associated with early recurrence of stage I non-small cell lung cancer. [33] However, gene silencing via hypermethylation is still a relatively important idea in the development of HNSCC, and little is known about the contribution of epigenetics to disease progression in HNSCC.

We systematically investigated *SST* and *SSTR1* promoter hypermethylation in primary HNSCC. To our knowledge, neither expression nor promoter hypermethylation of *SST* and *SSTR1* in HNSCC has been reported previously. Our results show that *SST* and *SSTR1* promoter hypermethylation occurs frequently in UM-SCC cell lines and primary tumors. The frequency of *SST* and *SSTR1* hypermethylation was extremely low in normal fibroblasts and keratinocytes and mucosal tissues. ROC curve analysis revealed that the AUROC values of *SST* and *SSTR1* methylation levels were significantly higher in the HNSCC patients. *SST* and *SSTR1* DNA methylation is a potential biomarker that could facilitate the differential diagnosis of HNSCC. An MSP survey of 100 tumor tissue samples demonstrated that hypermethylation of the *SST* promoter (81%) and *SSTR1* promoter (64%) occurred with a high frequency. Indeed, these rates were higher than methylation frequencies of other tumor suppressor loci such as *p16* (52%), *COL1A2* (48%), *H-cadherin* (43%) and *E-cadherin* (40%). A concurrent analysis showed that *SST* and *SSTR1* were completely methylated in 60 (60%) cases; another 25 (25%) cases were methylated at either *SST* or *SSTR1*, meaning that 85 (85%) cases were methylated at one or both *SST* and *SSTR1* promoters. Patients who had hypermethylation of both genes had lower survival rates than did patients without methylation of both genes; however, this was not statistically significant.

In multivariate logistic-regression analyses, adjusted for stage, age, sex, alcohol exposure, and smoking status, methylation of both *SST* and *SSTR1* was associated with an elevation in the odds of recurrence that was not significant. When both *SSTR1* and *galanin* were methylated in the primary tumors, the adjusted odds ratio for recurrence was higher than in tumors with other methylation patterns at these two loci. In another model of logistic regression with GMI, age, sex, alcohol exposure, and smoking status, *SSTR1* and *galanin* methylation has the highest odds ratio as an independent biomarker on its own.

Our study indicates that methylation of the promoter regions of neuropeptide genes in a resected HNSCC specimen is associated with tumor recurrence. The current method used to assess risk recurrence in patients with HNSCC is imprecise—indeed, half of such tumors recur after curative surgery. This information can be used to identify patients with high-risk HNSCC who may benefit from adjuvant therapy and close follow up observation after primary tumor resection. Our findings support the translation of such methylation markers into clinical practice, although additional prospective studies are required to validate these genes in larger populations of HNSCC patients.

## Supporting Information

S1 Fig Comparison of GMI among selected epidemiologic and clinical characteristics. GMI was calculated by taking the sum of gene promoter hypermethylation events (eight in this study; *p16*, *RASSF1A*, *E-cadherin*, *H-cadherin*, *MGMT*, *DAPK*, *DCC*, and *COL1A2*).
(EPS)

S2 Fig Kaplan-Meier survival curves for patients with HNSCC. Disease-free survival by **(A)** *TAC1* and *SSTR1* methylation status, **(B)** *galanin* and *SSTR1* methylation status, **(C)** *GALR2* and *SSTR1* methylation status, and **(D)** *GALR1* and *SSTR1* methylation status. Blue line, patients without methylation; red line, patients with methylation.
(EPS)

S3 Fig Odds ratios for recurrence based on multivariate logistic-regression adjusted for GMI (6–8 vs. <6), age (65 and older vs. <65), sex, alcohol exposure, and smoking status. (EPS)

S4 Fig Diagrammatic representation of *SST* and *SSTRs* expression analysis by quantitative-RT-PCR in UM-SCC cell lines and normal oropharyngeal samples (NOS). (EPS)

S1 Table *SST* and *SSTR1* genes methylation status in HNSCC primary samples. This is correlation with *p16*, *RASSF1A*, *E-cadherin*, *H-cadherin*, *MGMT*, *DAPK*, *DCC*, and *COL1A2* methylation status.
(DOCX)
