# Supplementary material for: Aberrant Methylation Inactivates Somatostatin and Somatostatin Receptor Type 1 in Head and Neck Squamous Cell Carcinoma
Source: PLoS One. 2015 Mar 3;10(3):e0118588. doi: 10.1371/journal.pone.0118588 (PMC4348545; doi:10.1371/journal.pone.0118588)
Supplement: S1 Table — This is correlation with p16, RASSF1A, E-cadherin, H-cadherin, MGMT, DAPK, DCC, and COL1A2 methylation status. (DOCX) [file pone.0118588.s005.docx]

Supplemental Table S1. *SST* and *SSTR1* Genes Methylation Status in HNSCC Primary Samples.

Characteristics (n=100)  *SST*  *SSTR1*

Present (81) Absent (19) P-value† Present (64) Absent (36) P-value†

*p16* methylation

Yes (52)

No (48)

*RASSF1A* methylation

Yes (20)

No (80)

*E-cadherin* methylation

Yes (40)

No (60)

*H-cadherin* methylation

Yes (43)

No (57)

*MGMT* methylation

Yes (24)

No (76)

*DAPK* methylation

Yes (32)

No (68)

*DCC* methylation

Yes (33)

No (67)

*COL1A2* methylation

Yes (48)

No (52)

†Fisher’s exact probability test

44

37

16

65

34

47

39

42

22

59

31

50

29

52

41

40

8

11

5

14

6

13

4

15

2

17

1

18

4

15

7

12

0.317

1

0.448

0.059

0.149

0.012

0.337

0.317

34

30

13

51

30

34

34

30

22

42

28

36

26

38

35

29

18

18

8

28

10

26

9

27

2

34

4

32

7

29

13

23

0.836

1

0.088

0.007

0.001

0.001

0.045

0.096
